# Supplementary material for: Toxicogenomic Effects of Dissolved Saxitoxin on the Early Life Stages of the Longfin Yellowtail (Seriola rivoliana)
Source: Mar Drugs. 2023 Nov 18;21(11):597. doi: 10.3390/md21110597 (PMC10671919; doi:10.3390/md21110597)
Supplement: Supplementary file 1 [file marinedrugs-21-00597-s001.zip › marinedrugs-2610856-supplementary.pdf]

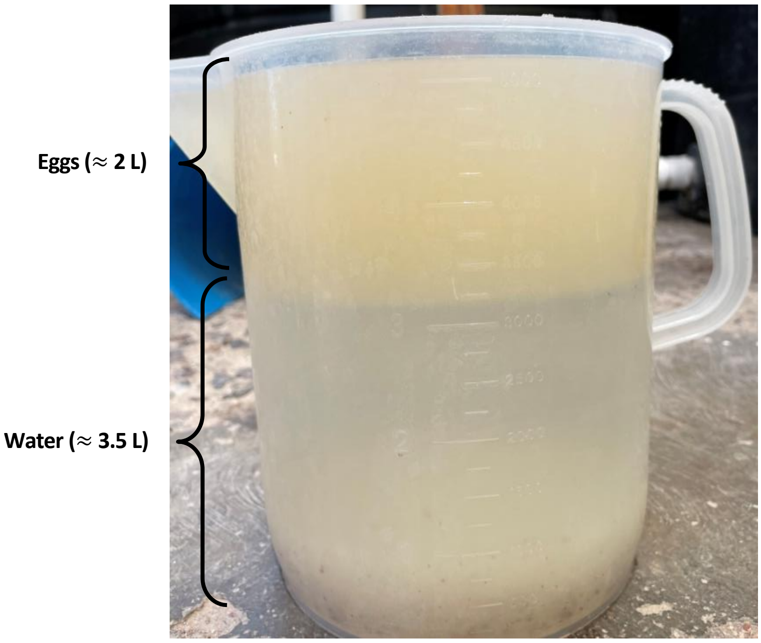


**Figure S1.** Spawning volume obtained from *S. rivoliana* broodstock reared by Kampachi Farms.

**Table S1.** Summary of the research on the effects of exposure to various concentrations of paralytic shellfish toxins (PSTs), including saxitoxin (STX) and gonyautoxin (GTX), in different fish species.

| Fish species | Life stage; Tissue | Toxin | Concentration | Exposure route and period | Effects | Reference |
|---|---|---|---|---|---|---|
| Capelin (*Mallotus villosus*); Atlantic herring *(Clupea harengus harengus)* | Capelin larvae aged 1, 2, 3, 5, 8, and 11 days; Herring larvae aged 1, 3, and 6 days; Herring post-larvae aged 10 and 15 days | PSTs (unspecified) | *A. tamarense* (2.5, 5, 10, and 15 x $10^5$ cells $L^{-1}$); zooplankton that had eaten *A. tamarens*e and extracted cellular content of *A. tamarense* | Oral ingestion and waterborne subacute exposure to dissolved PSTs | High mortality in fish larvae and early post-larvae either by direct or vectorial intoxication; Symptoms of paralysis; Insensitivity to dissolved toxin | [43] |
| Zebrafish *(Danio rerio)* | Embryos from 8 hpf to hatching; Larvae at 2, 4, and 6 dph | STX | 229 and 481 µg STX eq $L^{-1}$ | Dissolved STX subacute exposure (7 days) | Mortality; Growth retardation; Reduction in sensorimotor function as early as 48 hpf; Paralysis of larvae at 4 dpf; Severe edema of the eye, pericardium, and yolk sac in larvae 6 dpf | [23] |
| Pacific herring *(Clupea harengus pallasi)* | Larvae at 0, 4, and 11 dph | STX | 50, 400, 800, and 1,600 µg STX eq. $L^{-1}$ | Dissolved STX acute exposure (24 h) | Inhibition of sensorimotor function; Progressive age-specific increase in STX-induced paralysis; No STX-induced morphological abnormalities | [44] |

| | | | | | | |
|---|---|---|---|---|---|---|
| Killifish *(Fundulus heteroclitus)* | Adults; brain | STX | 75 and 150 ppb | Dissolved STX acute exposure (60 min) | Decrease in c-Fos protein expression; Behavioral alterations | [45] |
| Wolf fish *(Hoplias malabaricus)* | Adults; brain | 97% STX and 3% NeoSTX and GTX | 0.08 µg STX eq. 100 $g^{-1}$ tissue | Intraperitoneal inoculation; Subacute exposure (20 days) | Oxidative stress; Damage of DNA, proteins, and lipids | [46] |
| White seabream *(Diplodus sargus)* | Juveniles; Liver | N-sulfocarba-moyl and decarbamoyl toxins | 1.60 µg STX eq. $kg^{-1}$ body weight | Intracoelomic injection; Subacute exposure (2–6 days) | GST activity induction (phase II biotransformation); Chromosome breaks or loss; Segregational anomalies | [47] |
| Wolf fish (*Hoplias malabaricus*) | Adults; Liver and blood | 97% STX and 3% NeoSTX and GTX | 0.08 µg STX eq. 100 $g^{-1}$ tissue | Intraperitoneal inoculation; Subacute exposure (20 days) | Oxidative stress; No significant effect on the hepatosomatic index; Increase in hemoglobin concentration | [48] |
| Marine medaka (*Oryzias melastigma*) | Embryos | STX | 840 and 1260 µg. $L^{-1}$ | Dissolved STX subacute exposure (15 dph) | Abnormal growth; High neuron development; Decrease in VTG and vtg1 protein expression; Reduction in the yolk sac size; Up-regulation in glycolytic and lipid metabolic proteins; Increase in stress proteins | [49] |
| Longfin yellowtail *(Seriola rivoliana)* | Larvae at 0 and 3 dph | GTX2-3; dcGTX2-3; C1-2 | 0.1 µg STX eq. $mL^{-1}$ | Dissolved PSTs acute exposure (24 h) | High mortality; Increase in lipase activity and gene expression | [36] |
| Zebrafish *(Danio rerio)* | Embryos | STX | 0.05 and 0.1 µM | Dissolved STX subacute exposure (from 3 to 96 hpf) | Mortality; Oxidative stress; Apoptosis; DNA damage | [42] |

| | | | | | | |
|---|---|---|---|---|---|---|
| Zebrafish *(Danio rerio)* | Adults (males and females) | STX | 0.1 and 1 μg $L^{-1}$ | Dissolved STX chronic exposure (90 days) | Decrease in body weight and length: Oxidative stress; Immunosuppression; No significant effect on reproductive parameters | [50] |
| Longfin yellowtail (*Seriola rivoliana*) | Embryos at 8 and 26 hpf; Larvae at 44, 50, and 62 hpf | STX | 100 μg $L^{-1}$ | Dissolved STX subacute exposure (from 8 to 62 hpf) | High mortality; Stage-specific sensitivity to the toxin; Genetic regulation of NaV channels; HSP induction; Increase in adipogenesis and lipid metabolism; Regulation of MAP kinases and phosphatases activities | This study |

**Table S2.** Marine toxins, chemical structure and main mode of action in the intoxicated organism. Adapted from Vasconcelos et al. [22] and Guillotin & Delcourt [18].

| Toxin | Example of producing organism | Chemical type and structure | Target / mode of action |
|---|---|---|---|
| Saxitoxins | **Dinoflagellates**<br>*Alexandrium* spp<br>*Pyrodinium bahamense*<br>*Gymnodinium catenatum* | Alkaloid<br>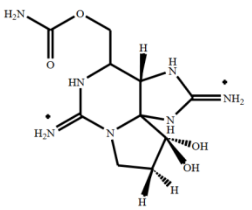 | $Na^+$ channels, site 1 of the $\alpha$ subunit / blockage<br>Ca2+ channels / blockage<br>K+ channels / blockage |
| Tetrodotoxin | **Bacteria**<br>*Vibrio* sp.<br>*Serratia marcescens* | Alkaloid<br>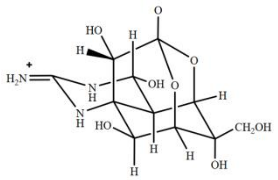 | $Na^+$ channels, site 1 of the $\alpha$ subunit / blockage |
| Brevetoxins | **Dinoflagellates**<br>*Karenia brevis* | Polyethers<br>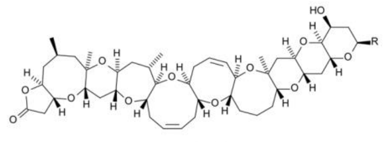<br>Brevetoxin A<br>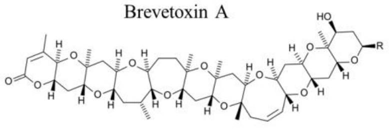<br>Brevetoxin B | $Na^+$ channels, site 5 of the $\alpha$ subunit / permanent activation |
| Ciguatoxin | **Dinoflagellates**<br>*Gambierdiscus* and *Fukuyoa* | Polycylic ethers<br>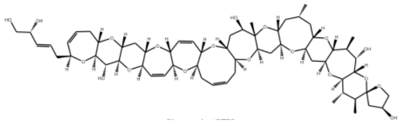 | $Na^+$ channels, site 5 of the $\alpha$ subunit / permanent activation<br>K+ channels / blockage |
| Okadaic acid | **Dinoflagellates**<br>*Dynophysis* and *Prorocentrum* | Polyethers<br>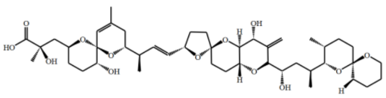 | Protein phosphatases / inhibition |
| Microcystin- | **Cyanobacteria**<br>*Microcystis aeruginosa* | Polyethers<br>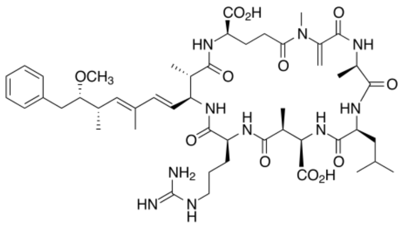 | Protein phosphatases / inhibition |
